# The impact of COVID‐19 on obesity services across Europe: A physician survey

**DOI:** 10.1111/cob.12474

**Published:** 2021-07-13

**Authors:** Katrin Nather, Fiachra Bolger, Laurie DiModica, Mary Fletcher‐Louis, Javier Salvador, François Pattou, Ulrik Haagen Panton, Ana‐Paula Cancino

**Affiliations:** ^1^ DRG Abacus, Part of Clarivate London UK; ^2^ Department of Endocrinology & Nutrition Clínica Universidad de Navarra Pamplona Spain; ^3^ CIBER Fisiopatología de la Obesidad y Nutrición (CIBEROBN) Instituto de Salud Carlos III Madrid Spain; ^4^ Univ Lille, CHU de Lille, Inserm Lille Pasteur Institute, Integrated Obesity Center Lille France; ^5^ Novo Nordisk North West Europe Pharmaceuticals A/S Copenhagen Denmark

**Keywords:** bariatric surgery, COVID‐19, Europe, obesity, obesity services, physician survey

## Abstract

Obesity is a risk factor for severe complications from coronavirus disease 2019 (COVID‐19). During the COVID‐19 pandemic in Spring 2020, many clinics and obesity centers across Europe were required to close. This study aimed to determine the impact of COVID‐19 on the provision of obesity services across 10 European countries via a survey of physicians (n = 102) specializing in treating persons with obesity (PwO). In total, 62–95 out of 102 physicians reported that COVID‐19 affected obesity‐related services, with cancellations/suspensions ranging from 50% to 100% across the 10 countries. Approximately 75% of cancellations/suspensions were provider‐ rather than patient‐initiated. A median increase of 20%–25% in waiting times was reported for most services across the countries. When services resume, 87 out of 100 physicians consider factors influencing down‐stream patient outcomes as the most relevant factors for prioritizing interventional treatment. Responses showed that 65 out of 102 and 36 out of 102 physicians believed it (highly) likely that a change in treatment guidance will occur to prioritize earlier interventional treatment for the management of PwO, by either using bariatric surgery or pharmacotherapy, respectively. Results from this study provide important learnings, such as opportunities for, and discrepancies in, the provision of alternative care in light of services cancellations or delays, which may be important for the future management of obesity, especially during future waves of COVID‐19 or other infectious pandemics.

**What is already known about this subject**

- Obesity is the most common comorbidity for patients infected with COVID‐19, and is a risk factor for severe complications from COVID‐19 infection
- Current European guidelines for the treatment of obesity recommend an integrated treatment of obesity with multidisciplinary provision of care, including diet, physical activity and behavioural therapy as first line treatment strategies and pharmacological treatment, and if indicated, bariatric or metabolic surgery as later line treatment approaches
- National lockdowns across Europe during the first wave of the COVID‐19 pandemic in Spring 2020 resulted in many non‐essential medical services being reduced or cancelled, however the impact on obesity services is unclear

**What this study adds**

- Findings from this small‐scale European physician survey demonstrate the impact of the COVID‐19 pandemic on obesity services in Spring 2020, and report most obesity services being cancelled, increased waiting time for patients, and a limited provision of care via alternative measures
- Results highlight how physicians anticipate the management of obesity may change in future, including prioritization of patients for pharmacological or surgical treatment, considering the risks associated with severe COVID‐19 infections
- Results from this study provide important learnings, such as opportunities for, and discrepancies in, the provision of alternative care in light of services cancellations or delays, which may be important for the future management of obesity during future waves of COVID‐19 or other infectious pandemics

**Abbreviations**
BMI: body mass index
COMs: centers for obesity management
COVID‐19: coronavirus disease 2019
EASO: European Association for the Study of Obesity
PwO: persons with obesity
SARS‐CoV‐2: severe acute respiratory syndrome coronavirus‐2
SI: supplementary information

## 1 INTRODUCTION

The coronavirus disease 2019 (COVID‐19) pandemic is caused by the severe acute respiratory syndrome coronavirus‐2 (SARS‐CoV‐2) and led to Europe entering a state of lockdown between February and May 2020, in which most countries cancelled elective medical services and surgical procedures to focus on treatment of patients with COVID‐19 and contain the virus.

The COVID‐19 pandemic affected European countries to varying degrees. Data from the Oxford COVID‐19 Government Response Tracker provide indications that “stay at home” requirements were implemented in March 2020 and ranged from localized recommendations (e.g., Denmark, Finland, Sweden and the Netherlands) to localized and national lockdowns (Belgium, France, Ireland, Portugal, Spain and the UK) throughout the first wave of the COVID‐19 pandemic.^1^ The European Society of Residents in Urology reports major elective surgeries being cancelled in France, Germany, Italy, Spain and the UK as a result of the COVID‐19 pandemic in Spring 2020,^2^ a scenario that was likely mirrored across Europe.

The cost of the disruption to medical services due to the pandemic has directly affected patients awaiting surgery. According to a study by the CovidSurg Collaborative, approximately 28.4 million elective surgeries were projected to be cancelled worldwide as a result of a 12‐week period of peak disruption during the first wave of the COVID‐19 pandemic.^3^ In the UK, the decision to postpone all non‐urgent elective operations for at least 3 months during the first wave of the pandemic (Spring 2020) was estimated to result in 516 000 surgeries being cancelled.^4^ This backlog has since increased with subsequent lockdowns.^5^

COVID‐19 is associated with several comorbidities including obesity, arterial hypertension, chronic lung disease, diabetes and cancer,^6^, ^7^, ^8^ with obesity being the most common comorbidity for patients infected with COVID‐19.^9^, ^10^ Additionally, evidence suggests that persons with obesity (PwO) who contract COVID‐19 have a higher risk of developing a severe illness due to the virus.^7^, ^8^, ^11^, ^12^, ^13^, ^14^, ^15^

Several explanations for why PwO are more susceptible to severe disease with COVID‐19 have been proposed, including impaired lung function,^16^ endothelial dysfunction,^14^ and inflammation.^17^, ^18^, ^19^ Other respiratory diseases, including influenza^20^, ^21^ and the H1N1 2009 strain of influenza,^19^, ^22^, ^23^ adversely affect PwO indicating that this population may be at heightened risk of developing severe illness due to COVID‐19.

Although younger age is considered to lower the risk for COVID‐19 disease severity, a growing body of evidence indicates that obesity in people aged <60 years is a risk factor associated with increased COVID‐19 disease severity and a risk factor for hospital admission and critical care. Patients with COVID‐19 and a BMI between 30–34 kg/m^2^ were 2.0 and 1.8 times more likely to be admitted to acute and critical care, respectively, compared with individuals with a BMI <30 kg/m^2^.^11^ Additionally, those with a BMI ≥25 kg/m^2^ had significantly higher odds of developing severe COVID‐19 disease requiring supplemental oxygen and intensive care admission.^24^ An inverse correlation between age and BMI has also been suggested, in which younger people admitted to hospital with COVID‐19 were more likely to have obesity, indicating that in populations with a high prevalence of obesity, COVID‐19 will affect younger populations more than initially reported.^25^

Alongside obesity being linked to severe incidence of COVID‐19, it has also been identified as an independent risk factor for high mortality in a large (>20 000 hospital inpatients) prospective observational cohort study in the UK between 6 February and 19 April 2020.^26^ Given the link between obesity and severe COVID‐19 disease and high mortality, it is imperative that PwO are adequately managed and treated. Any impact on the ability to adequately manage PwO may put patients at risk of developing and exacerbating comorbidities of obesity, and more severe infections of COVID‐19.

Current European guidelines for the treatment of obesity recommend an integrated treatment of obesity that may include diet, physical activity, pharmacological treatment, behavioural therapy and if indicated, bariatric or metabolic surgery, with multidisciplinary provision of care.^27^, ^28^

A recent publication by Rubino et al., provides comprehensive recommendations for prioritizing and managing surgical candidates and post‐operative patients during and after the peak of COVID‐19 waves, including pre‐operative screening, non‐surgical interventions and triaging of patients for priority surgery.^29^ When surgeries can resume, it has been proposed to stratify PwO based on a benefit/risk assessment with priority for those PwO whose need for surgery is high provided that the risk of morbidity due to the surgery is very low.^30^ Increased vigilance, priority on COVID‐19 detection and testing, and aggressive therapy are recommendations for PwO and COVID‐19 infections.^23^

Given the highly relevant link between COVID‐19 and obesity for healthcare services and public health bodies, it is essential to understand how services for PwO have been impacted by the pandemic. Here, we report the results of a quantitative survey with physicians involved in the treatment of PwO to understand the impact of COVID‐19 on obesity services across Europe in Spring 2020.

## 2 MATERIALS AND METHODS

The online survey (see supplementary information [SI]) was developed by DRG Abacus, Part of Clarivate, in collaboration with Novo Nordisk, and carried out by Sermo Inc. Physicians were recruited from a standing panel of specialists, and eligibility to participate in the study was established using the detailed screening criteria. Physicians were required to treat at least 10 PwO (defined as BMI ≥30 kg/m^2^) per month, and specialize in the management of PwO (e.g., endocrinologists, obesity specialists, and internal medicine physicians who indicated a subspecialty on obesity; primary care physicians were excluded from the survey). Participation in the survey was double‐blinded with the identity of the participants undisclosed. The study included 10–11 physicians from each of the following 10 European countries: Belgium, Denmark, Finland, France, Ireland, the Netherlands, Portugal, Spain, Sweden and the UK. A physician sample size of n = 10 was considered the minimum number of participants necessary to elucidate trends in practice patterns in the treatment of obesity due to the COVID‐19 pandemic. Surveys were carried out in English in the UK, Ireland, France and Spain and translated to local language for the remaining countries. Survey questions covered the following topic areas: referral of patients for bariatric surgery, cancellations of surgery or other weight loss services, and the management of patients whilst services are suspended and upon resumption. Respondents would only provide answers to services they are actively providing. All surveys were validated internally to minimize any potential ambiguity in the questions and approved for local use to meet industry compliance requirements by local Novo Nordisk affiliates prior to fielding. Physicians completed the online survey between May and June 2020 and were remunerated for their participation according to fair market value hourly rates specified by Novo Nordisk.

## 3 RESULTS

### 3.1 Respondent characteristics

In total, 102 physicians completed the survey. The physicians were endocrinologists (68 out of 102), obesity specialists (31 out of 102), and internal medicine specialists (3 out of 102). Prior to COVID‐19 restrictions, physicians provided obesity care in an outpatient setting of a hospital (75 out of 102), an office‐based/community practice setting (16 out of 102), by virtual consultations (9 out of 102), or an inpatient setting (2 out of 102). The physicians manage obesity with either non‐pharmacological disease management (100 out of 102), and/or unspecified pharmacotherapy (98 out of 102), and/or bariatric surgery (95 out of 102). Physician characteristics by country are provided in the SI (see Table S1).

### 3.2 Survey results

**FIGURE 1:**
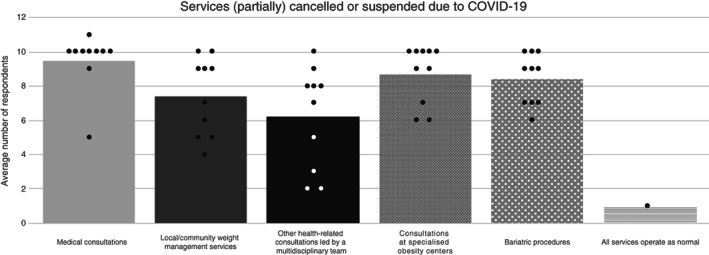
Impact of COVID‐19 on services. Average number of the total cohort of physicians reporting services (partially) cancelled or suspended due to COVID‐19. Columns represent the average number of physicians across the total cohort and the circles (●) represent the spread of the averaged country data per service. COVID‐19, coronavirus disease 2019

In total, 95 out of 102 physicians reported an impact of COVID‐19 on medical consultations, 87 out of 102 on consultations at specialized obesity centers, 84 out of 102 on bariatric procedures, 74 out of 102 on local/community weight management services, and 62 out of 102 on other health‐related consultations led by a multidisciplinary team. Responses by country are presented in Figure 1 and in the SI (Table S2). Across all countries, the COVID‐19 pandemic impacted all obesity services considered, with routine medical consultations by specialists and non‐specialists, and bariatric procedures reported as affected by the greatest number of physicians per country, whilst other health‐related consultations by a multi‐disciplinary team were reported to be affected by COVID‐19 by the least number of physicians per country.

Physicians were asked to provide the percentage of obesity appointments cancelled or suspended by the provider or patient due to the COVID‐19 pandemic. For all physicians, the percentage of cancellations or suspensions (hereafter referred to as cancellations) was 87% for local/community weight management services, 86% for bariatric procedures, 84% for consultations at speciality obesity centers, 80% for other health‐related consultations led by a multidisciplinary team, and 79% for medical consultations. The cancellations were mainly driven by the provider (57%–71%) and fewer cancellations were due to the patient (15%–23%). Individual country‐level data are provided in Table 1. Across the individual European countries, cancellations (50%–100%) were reported for the services considered. The highest percentage of cancellations of services were reported in France (97%–100%) and Spain (96%–98%), whereas the lowest number of cancellations for such services were reported in Ireland (58%–81%), Portugal (61%–80%) and Sweden (69–79%). Physicians in France reported cancellation of all bariatric procedures, mainly driven by the provider, during the COVID‐19 pandemic.

**TABLE 1:** Percentage cancellations for each service due to COVID‐19

		Belgium	Denmark	Finland	France	Ireland	Netherlands	Portugal	Spain	Sweden	UK	Total
Medical consultations	Total, % (n)	83 (10)	81 (10)	81 (10)	99 (9)	59 (10)	83 (10)	61 (10)	98 (5)	75 (10)	87 (11)	79 (95)
	Provider, %	56	55	60	67	42	63	51	61	49	72	57
	Patient, %	27	26	22	32	17	20	10	37	26	15	22
Local/community weight management services	Total, % (n)	86 (10)	86 (7)	86 (9)	98 (5)	81 (4)	85 (10)	80 (6)	96 (9)	69 (5)	90 (9)	87 (74)
	Provider, %	60	59	59	77	58	65	68	60	48	79	64
	Patient, %	26	28	26	21	24	20	12	36	21	11	23
Other health‐related consultations led by a multidisciplinary team	Total, % (n)	88 (2)	75 (3)	84 (8)	100 (5)	58 (7)	83 (9)	67 (10)	96 (8)	70 (2)	84 (8)	80 (62)
	Provider, %	68	45	62	78	40	62	53	63	40	71	59
	Patient, %	20	30	22	22	18	21	14	33	30	13	21
Consultations at specialized obesity centers	Total, % (n)	85 (10)	85 (10)	82 (10)	97 (6)	69 (10)	84 (9)	80 (9)	98 (6)	79 (10)	89 (7)	84 (87)
	Provider, %	64	62	60	82	48	58	63	62	55	80	62
	Patient, %	21	23	23	15	22	27	17	37	24	9	22
Bariatric procedures	Total, % (n)	91 (10)	86 (9)	82 (10)	100 (6)	57 (7)	86 (10)	94 (9)	97 (7)	79 (9)	89 (7)	86 (84)
	Provider, %	80	76	67	97	39	76	72	71	58	81	71
	Patient, %	12	11	15	3	18	11	23	26	22	8	15

Abbreviation: UK; United Kingdom.

Physicians reported an overall increase in the mean number of patients on waiting lists and waiting times for obesity management services. The median change in the number of patients on waiting lists and change in waiting times across the European countries are provided in the SI (Table S3) and Table 2 respectively. A median increase of 20%–25% in waiting times was observed for most services across the countries; however, a large variation was observed with physicians either reporting increases, decreases, or no change in numbers of patients on waiting lists and waiting times in each country.

**TABLE 2:** Median percentage change in waiting times of patients on waiting lists for obesity management services

		Belgium (n = 10)	Denmark (n = 10)	Finland (n = 10)	France (n = 10)	Ireland (n = 11)	Netherlands (n = 10)	Portugal (n = 10)	Spain (n = 10)	Sweden (n = 10)	UK (n = 11)
Medical consultations	Median (min, max)	25 (0, 45)	20 (20, 30)	25 (15, 45)	20 (−20, 80)	25 (−30, 40)	25 (20, 35)	5 (0, 30)	12.5 (−50, 80)	22.5 (−25, 50)	25 (−90, 75)
Local/community weight management services	Median (min, max)	25 (−15, 55)	–15 (−20, 15)	20 (−20, 40)	22.5 (−20, 60)	25 (−35, 40)	15 (−10, 55)	5 (0, 30)	15 (−20, 100)	17.5 (−15, 50)	25 (−90, 100)
Other health‐related consultations led by a multidisciplinary team	Median (min, max)	−27.5 (−30, 50)	25 (25, 25)	22.5 (−15, 25)	20 (−40, 60)	25 (−15, 40)	0 (−15, 15)	10 (0, 50)	20 (−50, 100)	25 (−30, 40)	20 (−90, 75)
Consultations at specialized obesity centers	Median (min, max)	−15 (−25, 50)	−15 (−30, 20)	−12.5 (−30, 30)	15 (−40, 80)	30 (−20, 40)	10 (−25, 25)	7.5 (0, 50)	12.5 (0, 80)	30 (−15, 60)	50 (−90, 75)
Bariatric procedures	Median (min, max)	−50 (−70, 50)	−10 (−70, 35)	−10 (−50, 35)	13.5 (−30, 100)	25 (10, 40)	−10 (−20, 25)	10 (0, 50)	10 (−50, 90)	25 (−70, 65)	30 (−100, 100)

*Note*: Negative numbers indicate a decrease in the waiting times of patients on waiting lists for obesity management services.

Abbreviation: UK; United Kingdom.

**FIGURE 2:**
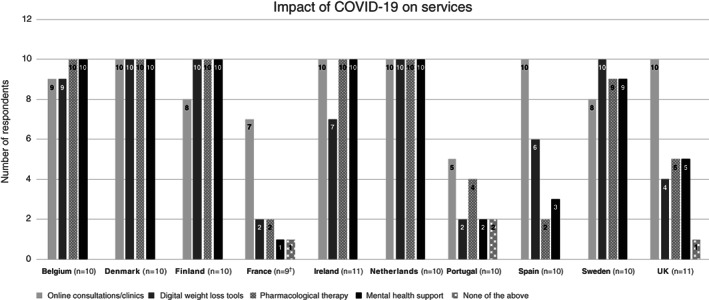
Alternative treatments provided for persons with obesity for whom surgical procedures or other in person weight management services have been cancelled or postponed. UK; United Kingdom. †One physician in France noted services operate as normal and is not included in this graph

Most physicians were providing at least one form of alternative treatments for PwO for whom in person services had been cancelled or postponed. The majority of physicians provided online consultations/clinics (87 out of 101), unspecified pharmacological therapy (72 out of 101), digital weight‐loss tools (70 out of 101), and mental health support (70 out of 101) as alternative treatments in the wake of the first wave of the COVID‐19 pandemic; however, four out of 101 physicians reported no provision of alternative services. Responses by country are presented in Figure 2. Almost all physicians from Belgium (9 out of 10–10 out of 10), Denmark (10 out of 10), Finland (8 out of 10–10 out of 10), the Netherlands (10 out of 10) and Sweden (8 out of 10–10 out of 10) reported the provision of at least one alternative treatment. Fewer physicians from France (1 out of 9–2 out of 9), Portugal (2 out of 10–4 out of 10), Spain (2 out of 10–6 out of 10) and the UK (4 out of 11–5 out of 11) reported a provision of alternative treatments, excluding online consultations.

**FIGURE 3:**
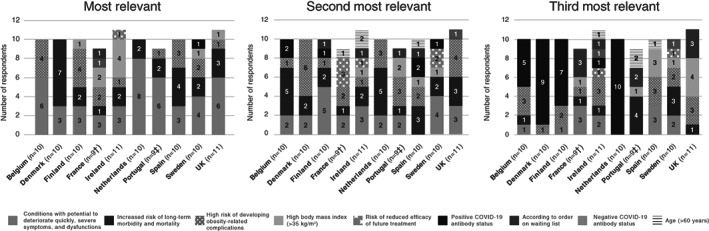
Physicians' expectations for the likely prioritization of persons with obesity for access to interventional treatment. UK; United Kingdom. †One physician in France noted services operate as normal and therefore was not asked this question. ‡One physician in Portugal selected “none of the above” and is not included in this graph

Physicians were asked to rank the three most relevant criteria for prioritizing PwO for interventional treatment including access to multi‐disciplinary teams, weight management clinics and bariatric procedures post‐COVID‐19 restrictions. Overall, responses showed that 87 out of 100 physicians consider factors influencing down‐stream patient outcomes, including conditions with the potential to deteriorate quickly, increased risk of long‐term morbidity and mortality, and high risk of developing obesity‐related complications as the most relevant factors for prioritizing PwO. A summary of responses by country is provided in Figure 3 and Table S4–Table S6. Of note, a positive COVID‐19 antibody status was considered among one of the third most relevant criteria for prioritization of patients by 31 out of 40 physicians in Belgium, Denmark, Finland and the Netherlands. However, only 13 out of 100 physicians considered age and the position of the patient on the waiting list among one of the third most relevant criteria for prioritizing patients.

**FIGURE 4:**
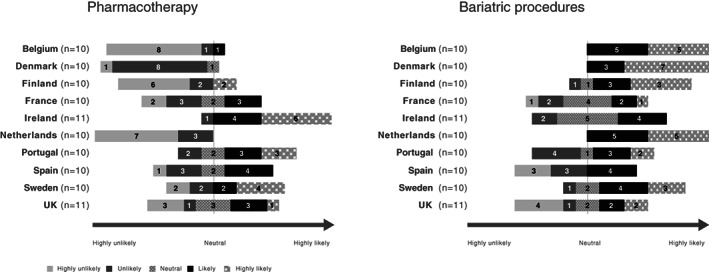
Diverging stacked bar chart presenting the likeliness for a change in treatment guidance to prioritize interventional treatment for persons with obesity in the future. UK; United Kingdom

Responses showed that 65 out of 102 and 36 out of 102 physicians believed it either “likely” or “highly likely” that there will be a change in treatment guidance for the management of PwO post‐COVID‐19 to prioritize bariatric surgery and pharmacological treatment, respectively (Figure 4). More than 6 out of 10 physicians in Belgium, Denmark, Finland and the Netherlands consider a change in treatment guidance in favour of pharmacotherapy to be either “unlikely” or “highly unlikely,” whilst a prioritization of bariatric procedures was considered more likely. In contrast, most physicians in Ireland consider a prioritization of pharmacotherapy in treatment guidance as highly likely (6 out of 11) compared with bariatric procedures (0 out of 11).

## 4 DISCUSSION

The results of this survey highlight the impact that the first wave of COVID‐19 in Spring 2020 has had on obesity‐related health services throughout Europe. In most countries considered in this survey, many non‐essential services were either cancelled or postponed to free up capacity, minimize the spread of infection, prevent COVID‐19 infections in vulnerable patient populations and focus healthcare resources on the treatment of patients with COVID‐19. An average of 61%–93% of obesity services were reported as having been either cancelled or postponed across 10 European countries. Personal and professional observations from numerous healthcare providers, including the clinical authors of this publication, suggest a complete cessation of non‐acute surgical procedures during most of the period February–June 2020. Hence, the findings from this survey may underestimate the actual impact of COVID‐19 on obesity services. In contrast, Sweden did not enter a full lockdown at the height of the European COVID‐19 pandemic in Spring 2020, and this may be reflected in fewer cancellations being reported for obesity services in Sweden.

This small‐scale survey suggests that COVID‐19 has likely impacted most obesity services throughout Europe. Routine medical consultations by specialists and non‐specialists, and bariatric procedures were the most affected, with other health‐related consultations by a multi‐disciplinary team being the least affected. Physicians reported both provider‐ and patient‐initiated cancellations, but with cancellations predominantly made by the provider. Cancellations initiated by the patient were approximately 20%–25% that of provider‐initiated cancellations per service suggesting that patients were also deprioritising their own treatment in the wake of the COVID‐19 pandemic.

The elective nature of bariatric surgery resulted in an almost complete cancellation of services across Europe. The International Federation for the Surgery of Obesity and Metabolic Disorders report a total of 134 487 bariatric surgeries performed between 2014 and 2018 across Western and Eastern Europe.^31^ Considering an average bariatric surgery cancellation rate of 86% reported across the countries surveyed in this study, this correlates with a cancellation of 7711 surgeries due to the COVID‐19 crisis between February and May 2020.

With bariatric surgery being the only interventional approach to the treatment of obesity in many countries, there is the potential for the cancellation of such procedures to substantially impact on the long‐term health of PwO, and as a result, increases in the numbers of patients on waiting lists and waiting times for PwO were observed for most services. Decreases in waiting list numbers and times observed in some countries suggest that no new patients entered the system due to a lack of consultations and referral to specialist services,^5^ or that the cancellation or suspension of surgeries and associated treatment methods due to COVID‐19, possibly resulted in the individual being removed from the waiting list. Woolf et al, suggest that during the COVID‐19 pandemic, there has been an increase in excess deaths possibly attributed to delayed care, exacerbation of chronic diseases and other factors in addition to the impact of COVID‐19.^32^ For example, limited screening, referral and diagnosis for cancer services during the pandemic could lead to a 20% increase in cancer deaths in the next 12 months.^33^ ^,^ ^34^ Obesity is a chronic, progressive and relapsing disease that requires long‐term treatment.^35^ Increased BMI is a major risk factor for disease‐related complications and comorbidities.^36^ Failure to treat or poor management of PwO leads to an increased risk of short‐term and long‐term complications and death.^37^ Although a high proportion of physicians specified that alternative management was provided to PwO whose services had been cancelled, a substantial number of patients may have been left with inadequate weight management support during this period, increasing their risk of disease‐related complications. A pooled analysis of five cross‐sectional surveys performed in Germany^38^ indicate that both total direct (medical) and indirect (incurred due to productivity losses) costs were significantly higher as BMI increased compared with normal weight persons, highlighting the future economic implications that inadequate obesity management during the COVID‐19 crisis may have on health systems, independent of the additional economic burden caused by COVID‐19 in the population.

This survey has demonstrated variations across countries with regards to the provision of alternative services to patients whose appointments had been cancelled. Specifically, physicians in France, Portugal, Spain and the UK appeared to provide alternative services to a lesser extent when compared with the other European countries. This may be reflective of physician attitudes towards provision of such services, the high number of cases of COVID‐19 in these countries with no capacity being available to provide patients with these alternative measures, or the infrastructure of the healthcare system, which emphasizes in‐person consultations with limited use of digital solutions for the provision of services.

Conditions that influence downstream outcomes, including conditions with the potential to deteriorate quickly, increased risk of long‐term morbidity and mortality, and high risk of developing obesity‐related complications, were highlighted in the survey as the most relevant factors for prioritization of PwO treatment post‐COVID‐19. This is unsurprising; given that these are criteria already employed by healthcare professionals when referring patients for bariatric surgery and are recommended in current treatment guidelines. Nevertheless, an increase of patients meeting these criteria may be expected, including both new patients whose diagnosis was delayed, and existing patients whose condition worsened during the COVID‐19 lockdown. Interestingly, a willingness is observed among physicians, particularly in Belgium, Denmark, Finland and the Netherlands, to consider a positive COVID‐19 antibody status when making treatment decisions. COVID‐19 testing is already employed in hospitals to confirm patients attending for procedures are not carrying an active infection of COVID‐19. In addition, a substantially lower risk of reinfection has been reported in individuals who tested positive for COVID‐19 antibodies.^39^ However, with countries embarking on large‐scale vaccination programmes and more people being vaccinated against COVID‐19, antibody testing is likely to be replaced in part by proof of a vaccination.

Considering the increased risk of severe disease and death due to COVID‐19 in PwO, physicians indicated that a shift to prioritize early interventional approaches, in particular bariatric procedures, and to a lesser extent pharmacotherapy, in treatment guidelines is anticipated in long‐term obesity treatment strategies post‐COVID‐19. It is to be expected that the demonstrated relationship between obesity and COVID‐19 severity will speed up the shift in public health policies acknowledging obesity as a disease, which should be addressed with preventative measures, and updated treatment guidance. Whilst pharmacotherapy could provide an alternative in the event of a resurgence of COVID‐19 and future lockdowns during which bariatric procedures may be cancelled, there are mixed responses with regards to the likelihood of pharmacotherapy being prioritized in treatment guidance. This is reflective of the current European anti‐obesity medication landscape, where access to licenced options and payer and physician attitudes to anti‐obesity drugs differs by country.

Following the first wave of COVID‐19, governments and health services are now recognizing the risks of obesity, and the associated potential for severe COVID‐19 infections.^40^ Accordingly, they have provided guidelines on the management of PwO during the COVID‐19 pandemic.^41^ Furthermore, targeted campaigns have been launched to communicate the benefits of weight loss in this vulnerable population, and to provide guidance on losing weight.

A statement from the European Association for the Study of Obesity (EASO) urges PwO to continue treatment and ensure awareness of all treatment and management options for their condition during the COVID‐19 pandemic.^42^ Contained within the release is an acknowledgement that although many clinics and obesity centers had to close during the pandemic, they are re‐opening under COVID‐safe procedures or have adapted to provide virtual consultations.

There are limitations to this survey. First, the small number of physicians per country and variations in locations within countries with different levels of prevalence of obesity and COVID‐19 that may not have been sampled, limit the generalizability of the study. Second, despite internal validation of questions to minimize ambiguity, there is the potential that questions may have been subject to varying interpretation by physicians, or physicians were not fully aware of the extent of lockdown on all services in which they practice, thereby leading to discrepancies in the responses within each country. Notably, most physicians suggested that they routinely prescribe pharmacotherapy, or have provided this as an alternative form of treatment for the management of obesity. This is surprising considering the status of the European anti‐obesity medication landscape. However, as the exact nature of the pharmacotherapy was not specified, it is unclear whether physicians were referring to anti‐obesity medication, and/or drugs used to treat and manage the symptoms and comorbidities of obesity. As a result of the limited approval of anti‐obesity medication in countries across Europe, there may have been a bias by the survey physicians towards the prioritization of bariatric surgery post‐pandemic considering that most services offered this measure as a treatment option. Lastly, information on the level of impact that individual respondents have on policy formation and decision making relating to changes in obesity management/prioritization resulting from the COVID‐19 pandemic, if any, is not available.

Despite these limitations, the results provide important implications for the future management of obesity, especially in the event of future waves of COVID‐19. First, the results highlight the broad impact of COVID‐19 on obesity services resulting in patients receiving limited or no treatment. This puts them at risk of future morbidity and mortality, and it will be important to establish measures that allow continuous management of patients, for example, via online consultations or digital approaches. Second, it has been demonstrated that not all countries are equally equipped or inclined to provide alternative treatment approaches to PwO when in‐person consultations are not possible, and that a concerted impetus to establish telemedicine and remote management of patients is necessary. Third, with bariatric surgery being the only interventional approach for the treatment of obesity and the risk of cancellations of procedures in future COVID‐19 waves, an alternative approach to achieve weight‐loss and prevent weight regain in PwO is required. Although physicians highlight that prioritization of bariatric surgery as an interventional approach in PwO post‐COVID‐19 is highly likely, benefit and risk to the patient will need to be considered. Consequently, these procedures may be at risk of cancellation during future waves of COVID‐19, leaving patients susceptible to increased complications, higher risk of death due to obesity and COVID‐19 infections.

A survey performed by the EASO across the EASO Collaborating Centers for Obesity Management (COMs) network reported findings similar to those reported in this publication.^43^ These findings include a substantial reduction in healthcare professionals available for obesity care, a complete suspension/reduction of in‐person outpatient visits in 98.6% of COMs during the COVID‐19 outbreak, a shift in obesity care services to a virtual alternative reported by 87.1% of COMs, and an obesity surgery cancellation/postponement rate of 96.5% (compared with a bariatric surgery cancellation rate of 86% reported in this publication). Cancellation of surgeries could potentially increase mortality risk in PwO with more advanced obesity and related comorbidities. Results from our survey corroborate those of the EASO survey wherein both surveys suggest that obesity care has been detrimentally affected during the COVID‐19 pandemic and indicate that innovative provision of obesity care via technology and telemedicine is occurring throughout Europe despite the COVID‐19 pandemic; however, the COVID‐19 pandemic is expected to accelerate this process.

Our study findings are further corroborated and extended by a recent survey from Public Health England investigating the impact of the COVID‐19 lockdown in March–June 2020 on patient behaviours and provision of services.^44^ In this report, 99% of surveyed adult service providers reported an impact of COVID‐19 on services, with 79% of Tier 2 (diet and lifestyle services) and 38% Tier 3 (specialist multidisciplinary care) services adapted or suspended. Similar to our findings, adaptation of service provisions included an increase in online consultations and support services, although it was noted that provision of these were highly variable across England. Furthermore, this report highlights that during the pandemic, 70%–80% of PwO report to have used food to manage their emotions with a change in physical activity due to the restrictions of movement, highlighting the strain COVID‐19 has placed on PwO and the relevant support services to effectively manage obesity.

In summary, this survey has shown that the first wave of the COVID‐19 pandemic in Spring 2020 has resulted in the cancellation of nearly all routine in‐person obesity services provided across Europe. Despite alternative services being provided to patients, due to the cancellation of in‐person services and bariatric procedures, their long‐term risk of morbidity and mortality may be impacted. With obesity being a risk factor for severe disease and mortality due to COVID‐19, physicians anticipate a change in treatment guidance to prioritize bariatric surgery, and potentially pharmacotherapy, as a long‐term strategy for the management of PwO post‐COVID‐19. This highlights the imperative nature of an earlier and more intensive treatment strategy that is warranted to manage the risk of comorbidities and severe COVID‐19 infections. The further waves of COVID‐19 hitting Europe during the time of writing of this manuscript, have demonstrated the persistence of those issues highlighted here, and the collective learnings from this survey remain of continued relevance for the improvement of obesity care post‐COVID‐19. As such, each country is required to apply their learnings and establish individual protocols to counteract the consequences and potential shortfall of services for PwO in future waves of COVID‐19.

## CONFLICT OF INTEREST

Katrin Nather, Fiachra Bolger, Laurie DiModica and Mary Fletcher‐Louis are employees of DRG Abacus, Part of Clarivate, and report that the study and manuscript preparation were funded by Novo Nordisk. Javier Salvador reports personal fees from Novo Nordisk, outside the submitted work. François Pattou has nothing to disclose. Ana‐Paula Cancino (Obesity Specialist in Medical Affairs Department) and Ulrik Haagen Panton report personal fees from Novo Nordisk, outside the submitted work, and are employees and stock option holders of Novo Nordisk. Novo Nordisk produces products to treat obesity.

## Supporting information


**Appendix** S1: Supporting Information
