# Supplementary material for: The impact of COVID‐19 on obesity services across Europe: A physician survey
Source: Clin Obes. 2021 Jul 13;11(5):e12474. doi: 10.1111/cob.12474 (PMC8420525; doi:10.1111/cob.12474)
Supplement: Supplementary file 1 — Appendix S1: Supporting Information [file COB-11-0-s001.pdf]

Supplementary information

Title

The impact of COVID-19 on obesity services across Europe: a physician survey

Authors

Katrin Nather,[1] Fiachra Bolger,[1] Laurie DiModica,[1] Mary Fletcher-Louis,[1] Javier Salvador,[2] François Pattou,[3] Ulrik Haagen Panton,[4] Ana-Paula Cancino[4]

[1]DRG Abacus, Part of Clarivate, Bicester, UK

[2]Department of Endocrinology & Nutrition, Clínica Universidad de Navarra, Pamplona, Spain; CIBER Fisiopatología de la Obesidad y Nutrición (CIBEROBN), Instituto de Salud Carlos III, Madrid, Spain

[3]Univ Lille, CHU de Lille, Inserm, Lille Pasteur Institute, Integrated Obesity Center, Lille 59000, France

[4]Novo Nordisk North West Europe Pharmaceuticals A/S, Copenhagen, Denmark

Corresponding author

Katrin Nather (katrin.nather@clarivate.com)

Address: DRG Abacus, Part of Clarivate, 6 Talisman Business Centre, Bicester, Oxfordshire, OX26 6HR

Telephone number: +44-1869-355-663

Survey

Adverse event reporting

Everything you will say during the market research remains anonymous, but if you provide any information on adverse events or product complaints, this will be reported to the client. Please note that our client registers information about the reported adverse events and product complaints for the sake of patient safety and to comply with current legislation. Your personal data will be entered and kept permanently in the client´s global safety database and will be treated confidentially. We are obliged to report safety information to Health Authorities in accordance with current legislation.

Are you happy to proceed with the survey on this basis?

☐	Yes, I would like to proceed and give my permission for my contact details to be passed on to the Drug Safety department of the company if an adverse event or product complaint is mentioned during the survey	
☐	Yes, I would like to proceed but do not wish for my contact details to be passed on to the Drug Safety department of the company if an adverse event or product complaint is mentioned by me during the survey	
☐	No, I don´t want to proceed and wish to end the survey here.	[PN: Terminate]

Screener

1) 1)[PN: SHOW FOR ALL EXCEPT UK] Are you currently providing obesity management care (weight loss or weight maintenance) to at least 10 patients/month living with obesity (BMI >30 kg/m^2)?

   [PN: SHOW FOR UK] Do you provide obesity management care (weight loss or weight maintenance) to at least 10 patients/month living with obesity (BMI >30 kg/m^2)

☐	Yes	
☐	No	[PN: Terminate]

2) What is your speciality? *Please select one*

☐	Endocrinologist	
☐	Obesity specialist	[PN: REMOVE OPTION FOR PORTUGAL AND SPAIN
☐	Internist	[PN: NETHERLANDS ONLY]
☐	Primary Care Physician	[PN: Terminate]
☐	Other	[PN: Terminate]

3) Which of the following treatment strategies do you currently routinely recommend or employ for obesity management? *You may select more than one answer – please tick all that apply*

☐	Non-pharmacological (e.g. weight management programmes, incl. diet, exercise, lifestyle interventions, and/or behavioural therapy)
☐	Pharmacotherapy
☐	Bariatric procedures
☐	Other (please specify)

4) Before COVID-19 restrictions, in which treatment setting did you principally manage your patients living with obesity? *Please select one*

☐	Inpatient
☐	Outpatient (hospital-based)
☐	Outpatient [PN: SHOW FOR ALL EXCEPT UK] (office-based)<br>[PN: SHOW FOR UK] (community practice)
☐	Virtual consultations
☐	Other (please specify) [PN: give text option]

Survey

1) How has COVID-19 impacted on the services provided to patients living with obesity? *Please select all that apply*

☐	At least some medical consultations (by Primary Care Physician, Endocrinologist, other HCPs not specialised in obesity management) have been cancelled/suspended
☐	At least some local/community weight management services (e.g. lifestyle interventions provided by weight management programmes in the community or workplace) have been cancelled/suspended
☐	At least some other health-related consultations led by a multidisciplinary team (e.g. nutritionist, psychologist, physiotherapist, endocrinologist) have been cancelled/suspended
☐	At least some consultations at specialised obesity centres have been cancelled/suspended
☐	At least some bariatric procedures have been cancelled/suspended
☐	All services operate as normal [PN: If this is selected, no other options can be selected]

2) [PN: Only show those options from options 1-5 ticked in Q1; if option 6, skip to Q3] For each service, please provide an estimate of the percentage cancellations by the provider and by the patient. *These would only sum to 100% if all consultations were cancelled*

**Service**	**Total (%)**	**Provider (%)**	**Patient (%)**
Medical consultations (by Primary Care Physician, Endocrinologist, other HCPs not specialised in obesity management) [PN: Sum of provider+patient column must equal total column]			
Local/community weight management services (e.g. lifestyle interventions provided by weight management programmes in the community or workplace) [PN: Sum of provider+patient column must equal total column]			
Other health-related consultations led by a multidisciplinary team (e.g. nutritionist, psychologist, physiotherapist, endocrinologist) [PN: Sum of provider+patient column must equal total column]			
Consultations at specialised obesity centres [PN: Sum of provider+patient column must equal total column]			
Bariatric procedures [PN: Sum of provider+patient column must equal total column]			

3) On average, what is the percentage change in the number of patients on **waiting lists** for the obesity management services listed below from before COVID-19 restrictions (1st February 2020) up to now (May 2020)? *Please provide one answer per row*

Service	% increase	No change	% decrease
Medical consultations (by Primary Care Physician, Endocrinologist, other HCPs not specialised in obesity management) [PN: only one answer per row is possible]			
Local/community weight management services (e.g. lifestyle interventions provided by weight management programmes in the community or workplace) [PN: only one answer per row is possible]			
Other health-related consultations led by a multidisciplinary team (e.g. nutritionist, psychologist, physiotherapist, endocrinologist) [PN: only one answer per row is possible]			
Consultations at specialised obesity centres [PN: only one answer per row is possible]			
Bariatric procedures [PN: only one answer per row is possible]			

4) On average, what is the percentage change in the **waiting times** for patients living with obesity being referred to the obesity management services listed below from before COVID-19 restrictions (1st February 2020) up to now (May 2020). *Please provide one answer per row*

**Service**	**% increase**	**No change**	**% decrease**
Medical consultations (by Primary Care Physician, Endocrinologist, other HCPs not specialised in obesity management) [PN: only one answer per row is possible]			
Local/community weight management services (e.g. lifestyle interventions provided by weight management programmes in the community or workplace) [PN: only one answer per row is possible]			
Other health-related consultations led by a multidisciplinary team (e.g. nutritionist, psychologist, physiotherapist, endocrinologist) [PN: only one answer per row is possible]			
Consultations at specialised obesity centres [PN: only one answer per row is possible]			
Bariatric procedures [PN: only one answer per row is possible]			

5) [PN: Skip is Q1 option 6 selected] Are any of your patients living with obesity for whom surgical procedures or other IN PERSON weight management services have been cancelled or postponed, receiving alternative treatments? *Please select all that apply*

☐	Online consultations/clinics
☐	Supply of digital weight loss tools, such as apps
☐	Pharmacological therapy
☐	Mental health support (e.g. stress, depression, anxiety)
☐	None of the above [PN: If this is selected, no other answers can be selected]
☐	Other (please specify) [PN: give text option]

6) [PN: Skip is Q1 option 6 selected] When obesity services are set to resume, how do you anticipate patients living with obesity will be prioritised for interventional treatment, such as access to multi-disciplinary teams, weight management clinics and bariatric procedures? *Please rank the three most relevant criteria, where 1 = most relevant, 2 = second most relevant, 3 = third most relevant*

**Criteria**	**Rank**
Conditions with potential to deteriorate quickly, severe symptoms and dysfunctions (e.g. dysphagia, deficiencies, sleep apnoea)	
Increased risk of long-term morbidity and mortality	
High risk of developing obesity-related complications	
High body mass index (>35 kg/m^2)	
Risk of reduced efficacy of future treatment	
Positive COVID-19 antibody status	
Negative COVID-19 antibody status	
According to order on waiting list	
Age (>60 years)	
None of the above [PN: If this is selected, no other answers can be selected]	
Other (please specify) [PN: give text option]	

7) With obesity considered one of the major risk factors for more severe disease and mortality due to COVID-19, in your opinion, how likely will there be a change in treatment guidance to prioritise interventional treatment (i.e. pharmacological treatment, bariatric surgery) for patients with obesity in the future? *Please provide your answer on a scale of 1-5, where 1 = highly unlikely and 5 = highly likely, considering both pharmacotherapy and bariatric procedures*

Pharmacotherapy	☐ 1	☐ 2	☐ 3	☐ 4	☐ 5
Bariatric procedures	☐ 1	☐ 2	☐ 3	☐ 4	☐ 5

Survey results

Table S1:Physician characteristics

			Speciality			Treatment strategies			Treatment setting			
	Number of physicians	Obesity management care (≥10 patients/month living with obesity (BMI>30kg/m²)	Endocrinologist	Obesity specialist	Internist	Non-pharmacological	Pharmacotherapy (unspecified)	Bariatric procedures	Inpatient	Outpatient	Outpatient (office-based/community practice)	Virtual consultations
Belgium (n=10)	10	10	5	5	–	10	10	10	1	9	–	–
Denmark (n=10)	10	10	5	5	–	10	10	10	–	10	–	–
Finland (n=10)	10	10	5	5	–	10	10	10	–	10	–	–
France (n=10)	10	10	9	1	–	10	7	10	1	4	3	2
Ireland (n=11)	11	11	5	6	–	10	11	7	–	3	5	3
Netherlands (n=10)	10	10	4	3	3	10	10	10	–	10	–	–
Portugal (n=10)	10	10	10	–	–	9	10	9	–	10	–	–
Spain (n=10)	10	10	10	–	–	10	10	10	–	5	3	2
Sweden (n=10)	10	10	5	5	–	10	10	9	–	4	5	1
UK (n=11)	11	11	10	1	–	11	10	10	–	10	–	1
**Total**	**102**	**102**	**68**	**31**	**3**	**100**	**98**	**95**	**2**	**75**	**16**	**9**

Abbreviations: BMI; Body mass index; UK; United Kingdom.

Table S2: Impact of COVID-19 on services

	Medical consultations	Local/community weight management services	Other health-related consultations led by a multidisciplinary team	Consultations at specialised obesity centres	Bariatric procedures	All services operate as normal
Belgium (n=10)	10	10	2	10	10	–
Denmark (n=10)	10	7	3	10	9	–
Finland (n=10)	10	9	8	10	10	–
France (n=10)	9	5	5	6	6	1
Ireland (n=11)	10	4	7	10	7	–
Netherlands (n=10)	10	10	9	9	10	–
Portugal (n=10)	10	6	10	9	9	–
Spain (n=10)	5	9	8	6	7	–
Sweden (n=10)	10	5	2	10	9	–
UK (n=11)	11	9	8	7	7	–
**Total**	**95**	**74**	**62**	**87**	**84**	**1**
**Percentage**	**93.1**	**72.5**	**60.8**	**85.3**	**82.4**	**1.0**

Abbreviations: UK; United Kingdom.

Table S3: Median percentage change in the number of patients on waiting lists for obesity management services

		Belgium (n=10)	Denmark (n=10)	Finland (n=10)	France (n=10)	Ireland (n=11)	Netherlands (n=10)	Portugal (n=10)	Spain (n=10)	Sweden (n=10)	UK (n=11)
Medical consultations	Median, % (min, max)	25 (10, 65)	20 (20, 55)	31.5 (20, 65)	20 (–90, 85)	25 (–40, 50)	25 (15, 55)	10 (0, 50)	5 (–50, 50)	22.5 (20, 50)	20 (–60, 90)
Local/community weight management services	Median, % (min, max)	22.5 (–25, 35)	32.5 (–15, 35)	20 (–25, 55)	2.5 (–75, 90)	20 (–40, 40)	–10 (–10, 40)	12.5 (–50, 60)	10 (–50, 70)	25 (–20, 60)	25 (–70, 85)
Other health-related consultations led by a multidisciplinary team	Median, % (min, max)	–15 (–20, 25)	–15 (–20, 25)	17.5 (–20, 70)	17.5 (–50, 100)	20 (–30, 40)	15 (–15, 20)	10 (–50, 50)	0 (–50, 100)	-15 (–35, 45)	30 (–70, 100)
Consultations at specialised obesity centres	Median, % (min, max)	–20 (–20, 30)	15 (15, 25)	–12.5 (–20, 30)	10 (–50, 100)	15 (–40, 50)	12.5 (–10, 25)	5 (–30, 50)	0 (–80, 50)	25 (–25, 50)	20 (–90, 100)
Bariatric procedures	Median, % (min, max)	–12.5 (-70, 70)	–10 (–15, 75)	–12.5 (–35, 35)	0 (–100, 100)	0 (–50, 40)	–12.5 (–25, -10)	5 (–10, 80)	–7.5 (–95, 80)	–15 (–25, 75)	20 (–100, 100)

Abbreviations: UK; United Kingdom.
Negative numbers indicate a decrease in the number of patients on waiting lists for obesity management services.

Table S4: Most relevant choices for the criteria for prioritisation of interventional treatment

	Most relevant								
	Conditions with potential to deteriorate quickly, severe symptoms and dysfunctions	Increased risk of long-term morbidity and mortality	High risk of developing obesity-related complications	High BMI (>35 kg/m^2)	Risk of reduced efficacy of future treatment	Positive COVID-19 antibody status	Negative COVID-19 antibody status	According to order on waiting list	Age (>60 years)
Belgium (n=10)	6	–	4	–	–	–	–	–	–
Denmark (n=10)	3	7	–	–	–	–	–	–	–
Finland (n=10)	3	2	4	1	–	–	–	–	–
France (n=10)	2	1	2	2	–	–	1	1	–
Ireland (n=11)	3	2	1	4	1	–	–	–	–
Netherlands (n=10)	8	2	–	–	–	–	–	–	–
Portugal (n=10)	6	2	1	–	–	–	–	–	–
Spain (n=10)	3	4	3	–	–	–	–	–	–
Sweden (n=10)	4	2	2	1	–	1	–	–	–
UK (n=11)	6	3	1	1	–	–	–	–	–
**Total**	**44**	**25**	**18**	**9**	**1**	**1**	**1**	**1**	**0**
**Rank**	**1**	**2**	**3**	**4**	**5**	**5**	**5**	**5**	**9**
**Percentage**	**43.1**	**24.5**	**17.6**	**8.8**	**1.0**	**1.0**	**1.0**	**1.0**	**0**

Abbreviations: BMI; Body mass index; UK; United Kingdom.

Table S5: Second most relevant choices for the criteria for prioritisation of interventional treatment

	Second most relevant								
	Conditions with potential to deteriorate quickly, severe symptoms and dysfunctions	Increased risk of long-term morbidity and mortality	High risk of developing obesity-related complications	High BMI (>35 kg/m2)	Risk of reduced efficacy of future treatment	Positive COVID-19 antibody status	Negative COVID-19 antibody status	According to order on waiting list	Age (>60 years)
Belgium (n=10)	2	5	1	–	–	2	–	–	–
Denmark (n=10)	2	2	6	–	–	–	–	–	–
Finland (n=10)	5	2	1	–	–	1	–	1	–
France (n=10)	2	1	2	–	3	–	–	–	1
Ireland (n=11)	3	2	1	1	1	–	–	1	2
Netherlands (n=10)	2	5	3	–	–	–	–	–	–
Portugal (n=10)	2	1	3	2	–	–	–	1	–
Spain (n=10)	–	3	2	1	–	2	–	1	1
Sweden (n=10)	4	2	–	1	2	1	–	–	–
UK (n=11)	3	3	4	–	–	–	1	–	–
**Total**	**25**	**26**	**23**	**5**	**6**	**6**	**1**	**4**	**4**
**Rank**	**2**	**1**	**3**	**6**	**4**	**4**	**9**	**7**	**7**
**Percentage**	**24.5**	**25.5**	**22.5**	**4.9**	**5.9**	**5.9**	**1.0**	**3.9**	**3.9**

Abbreviations: BMI; Body mass index; UK; United Kingdom.

Table S6: Third most relevant choices for the criteria for prioritisation of interventional treatment

	Third most relevant								
	Conditions with potential to deteriorate quickly, severe symptoms and dysfunctions	Increased risk of long-term morbidity and mortality	High risk of developing obesity-related complications	High BMI (>35 kg/m2)	Risk of reduced efficacy of future treatment	Positive COVID-19 antibody status	Negative COVID-19 antibody status	According to order on waiting list	Age (>60 years)
Belgium (n=10)	1	1	3	–	–	5	–	–	–
Denmark (n=10)	–	–	1	–	–	9	–	–	–
Finland (n=10)	1	–	2	–	–	7	–	–	–
France (n=10)	3	1	1	1	–	–	–	3	–
Ireland (n=11)	2	1	3	–	1	1	1	1	1
Netherlands (n=10)	–	–	–	–	–	10	–	–	–
Portugal (n=10)	–	4	–	1	–	–	–	2	2
Spain (n=10)	3	–	3	3	–	–	–	–	1
Sweden (n=10)	2	3	2	1	1	–	1	–	–
UK (n=11)	–	1	3	4	–	–	–	3	–
**Total**	**12**	**11**	**18**	**10**	**2**	**32**	**2**	**9**	**4**
**Rank**	**3**	**4**	**2**	**5**	**8**	**1**	**8**	**6**	**7**
**Percentage**	**11.8**	**10.8**	**17.6**	**9.8**	**2.0**	**31.4**	**2.0**	**8.8**	**3.9**

Abbreviations: BMI; Body mass index; UK; United Kingdom.
